# Prediction of out-of-field recurrence after chemoradiotherapy for cervical cancer using a combination model of clinical parameters and magnetic resonance imaging radiomics: a multi-institutional study of the Japanese Radiation Oncology Study Group

**DOI:** 10.1093/jrr/rrab104

**Published:** 2021-12-03

**Authors:** Hitoshi Ikushima, Akihiro Haga, Ken Ando, Shingo Kato, Yuko Kaneyasu, Takashi Uno, Noriyuki Okonogi, Kenji Yoshida, Takuro Ariga, Fumiaki Isohashi, Yoko Harima, Ayae Kanemoto, Noriko Ii, Masaru Wakatsuki, Tatsuya Ohno

**Keywords:** cervical cancer, chemoradiotherapy, MRI, out-of-field recurrence (OFR), prediction, radiomics

## Abstract

We retrospectively assessed whether magnetic resonance imaging (MRI) radiomics combined with clinical parameters can improve the predictability of out-of-field recurrence (OFR) of cervical cancer after chemoradiotherapy. The data set was collected from 204 patients with stage IIB (FIGO: International Federation of Gynecology and Obstetrics 2008) cervical cancer who underwent chemoradiotherapy at 14 Japanese institutes. Of these, 180 patients were finally included for analysis. OFR-free survival was calculated using the Kaplan–Meier method, and the statistical significance of clinicopathological parameters for the OFR-free survival was evaluated using the log-rank test and Cox proportional-hazards model. Prediction of OFR from the analysis of diffusion-weighted images (DWI) and T2-weighted images of pretreatment MRI was done using the least absolute shrinkage and selection operator (LASSO) model for engineering image feature extraction. The accuracy of prediction was evaluated by 5-fold cross-validation of the receiver operating characteristic (ROC) analysis. Para-aortic lymph node metastasis (*p* = 0.003) was a significant prognostic factor in univariate and multivariate analyses. ROC analysis showed an area under the curve (AUC) of 0.709 in predicting OFR using the pretreatment status of para-aortic lymph node metastasis, 0.667 using the LASSO model for DWIs and 0.602 using T2 weighted images. The AUC improved to 0.734 upon combining the pretreatment status of para-aortic lymph node metastasis with that from the LASSO model for DWIs. Combining MRI radiomics with clinical parameters improved the accuracy of predicting OFR after chemoradiotherapy for locally advanced cervical cancer.

## INTRODUCTION

The treatment options for cervical cancer include surgery, radiotherapy and chemotherapy; the choice of treatment from amongst these strategies is currently being determined mainly based on the stage of the disease and pathological diagnosis. However, the risk of local recurrence or metastasis for the same histological type of cancer in the same stage vary from case to case. The ability to predict the effects of radiotherapy, the risk of lymph node metastases and hematologic metastasis prior to treatment allows for the selection of a treatment method that is appropriate for each case. Additionally, personalized treatment is expected to improve the treatment outcomes. Scheduled adjuvant chemotherapy for locally advanced cervical cancer that is predicted to have a high risk of subsequent out-of-field recurrence (OFR) may improve treatment outcomes. Lymph node metastasis and histology have been reported to be predictive factors of OFR after definitive radiotherapy for cervical cancer [1, 2]. Development of predictive models with additional biomarkers may further improve the accuracy of the prediction.

Image analysis using engineering feature extraction is a computer-based technique for extracting features of images that cannot be detected by the human eye [3, 4]. Its usefulness as a biomarker has been reported in many clinical oncology studies [5]. In gynecologic oncology, the usefulness of image analysis of ^18^F-fluorodeoxyglucose positron emission tomography (^18^F-FDG-PET) or magnetic resonance imaging (MRI) using engineering feature extraction to estimate pathological findings (lymphatic invasion) and predict therapeutic effects has been reported [6–13]. However, all of these studies on cervical cancer have been limited to retrospective studies with small sample sizes from a single institute [8, 9, 14–16].

In this study, we retrospectively assessed the accuracy of a predictive model for OFR of cervical cancer in stage IIB (FIGO: International Federation of Gynecology and Obstetrics 2008) after definitive concurrent chemoradiotherapy (CCRT) in a cohort of patients treated at 14 institutes in Japan, using a combination model of pretreatment clinical parameters and MRI radiomics.

## MATERIALS AND METHODS

### Patient clinical information

The working group of the gynecological tumor committee of the Japanese Radiation Oncology Study Group (JROSG) conducted a survey study. Fourteen radiation oncology centers in Japan belonging to the JROSG participated in the present study. The Institutional Review Boards of all 14 institutes approved the study. We reviewed the radiation oncology records of the 204 consecutive patients with stage IIB (FIGO 2008) cervical cancer who underwent radical CCRT between January 2011 and December 2015. Most patients with past histories of malignancy were excluded from the study; however, patients who were treated for a malignant tumor but who did not develop a recurrence more than five years after completion of treatment were included. Clinical information on the patients and tumors, treatment methods and follow-up information were derived from the hospital information and radiation oncology information system of each of the institutions. Diffusion-weighted images (DWIs) (b value = 800–1000 mm^2^/s) and T2-weighted images (T2WIs) of MRIs within a month before the initiation of CCRT were obtained from the picture archives and communication systems of each institution.

OFR was defined as a hematologic metastasis or lymph node metastasis of outside of the field of irradiation. OFR within three years after the initiation of CCRT was defined as a predictive event.

### Patient population

We enrolled a total of 204 patients in this study. From these, we included 180 patients who had been followed-up for more than three years without OFR and patients who developed OFR within three years, excluding seven patients with MRI determined to be inappropriate due to insufficient coverage or artifacts. The characteristics of the 180 patients and tumors are summarized in Table 1.

**Table 1:** Patient characteristics

Age	Median (range)	56 (29–80)
Performance status	0	132
	1	45
	2	3
Pathological diagnosis	Squamous cell carcinoma	167
	Adenocarcinoma	7
	Adenosquamous carcinoma	3
	Neuroendocrine tumor	1
	Carcinosarcoma	1
	Small cell carcinoma	1
Maximum diameter of primary tumor	Median (range), cm	5.1 (2.2–17.2)
Volume of primary tumor	Median (range), ml	33.0 (3.0–557.4)
No. of pelvic lymph node metastasis	≧3	19
	1–2	49
	No	112
Common iliac lymph node metastasis	Yes	19
	No	161
Para-aortic lymph node metastasis	Yes	21
	No	159

### Chemoradiotherapy

All patients underwent external beam radiotherapy (EBRT) and high-dose-rate (HDR) brachytherapy (BT). EBRT was performed using three-dimensional conformal radiotherapy in all patients. For pelvic EBRT, 6–15 MV X-rays were applied using anteroposterior opposed fields or a four-field box technique for a daily fraction of 1.8 or 2.0 Gy delivered five times per week, for a total dose of 45–60.6 Gy (median 50 Gy). Central shielding was applied with 10–48 Gy (median 32 Gy) for 175 patients (97.2%). Twenty-nine patients had extended fields of radiation that included para-aortic lymph node areas, including nine patients with prophylactic irradiation. For intra-cavitary BT, a remote after-loading system with HDR ^192^Ir sources was used in 176 patients (97.8%), and with ^60^Co sources was used in four patients (2.3%). In the HDR intra-cavitary BT, a total dose of 12–36.1 Gy (median 22.4Gy) was delivered in 2–5 fractions at point A.

Chemotherapy was administered to all patients, in the concurrent setting. Weekly cisplatin at a dose of 30–40 mg/m^2^ × 1–7 times (median, 5 times) was administered to 133 patients, daily cisplatin to 15 patients and other regimens to 32 patients (Table 2). No patient underwent adjuvant chemotherapy.

**Table 2:** Treatment methods

External-beam radiotherapy		
Radiation field	Whole pelvis	151
	Whole pelvis + Para-aortic region	29
X-rays	6 MV	9
	10 MV	167
	15 MV	4
Whole pelvic irradiation		
Total dose (Gy)	Median (range)	50.0 (45.0–60.6)
Fraction number	Median (range)	25 (25–38)
Central shielding	Yes	175
	No	5
Boost therapy to LN metastasis		
	Yes	51
	No	129
Brachytherapy		
Source	^192^Ir	176
	^60^Co	4
Total dose at point A (Gy)	Median (range)	22.4 (12.0–36.1)
Fraction number	2	1
	3	54
	4	123
	5	2
Concurrent chemotherapy		
Weekly CDDP		133
Daily CDDP		15
Others		32

*LN =* lymph node

### Engineering feature extraction of magnetic resonance imaging and prediction model of out-of-field recurrence

In the first step of engineering feature extraction, segmentation of the primary lesion was performed by certified radiation oncologists and radiologists. In order to reduce inter-observer variation in segmentation, primary tumor contouring was performed in such a way that one radiologist and two radiation oncologists were in agreement. Then, after the resampling process (with a voxel of 2 × 2 × 2 mm^3^), shape-size, histogram and texture features were sequentially extracted from the original image and the processed images after a 3-dimensional wavelet transformation. A more detailed engineering feature extraction method can be found in Ref. [4]. In the present study, voxel values from nine segmented images (eight wavelet-transformed images and the original image) were normalized by two ways, a 0–1 scale (or min–max normalization) and z-score scale (or z-score normalization). A total of 458 features were obtained per image sequence. The above feature extraction method was performed semiautomatically using a MATLAB (2016b; MathWorks, Natick, MA) programming tool for radiomics analysis (based on https://github.com/mvallieres/radiomics/). In prediction of OFR, the Ridge model was employed for pretreatment clinical parameters, whereas the least absolute shrinkage and selection operator (LASSO) model was employed for engineering features and for the combination of pretreatment clinical parameters and engineering features, with the OFR-free survival rate at three years after initiation of CCRT as the output, and where the hyperparameter was determined by leave-one-out analysis in the training cohort. The data weight in the loss function was corrected because of the imbalanced data.

**Fig. 1:**
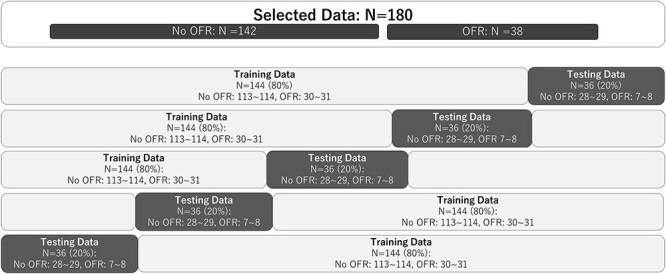
Five-fold cross validation that swaps the training data and testing data N, number; OFR, out-of-field recurrence.

Furthermore, we used Ridge model, Naïve bayes model and Random Forest (Gini index) model as other machine learning models of DWIs of MRI and analyzed them using a different feature selection method from the LASSO model as follows. The patients were classified into two groups based on the median value of each feature. Kaplan–Meyer analysis was performed in the two groups, and the features were arranged in order of decreasing *p*-value. The features with high correlation (>0.7) with the top features were excluded, and finally the top 10 features were selected.

### Statistical analysis

**Fig. 2:**
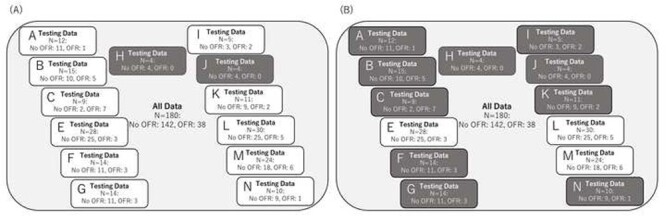
Classification according to the institute. (A) One institute was used as test data and the rest as training data. However, since there were no OFR cases in institutes H and J, these were always considered as training data. (B) Three institutions out of 13 institutions included more than 20 patients, and one of these institutions was selected as the test cohort and the rest 12 institutions were used in the model development with 5-fold cross validation.

Survival and local control periods were defined as the periods from the date of the first CCRT fraction to the date of the events. Survival and local control rates were calculated using the Kaplan–Meier method, and the significance of differences was examined using the Log-rank test. Cox proportional hazards model was used for multivariate analysis in the step to select statistically significant pretreatment clinical factors for prediction of OFR. The accuracy of predicting the OFR from the analysis of pretreatment clinical parameter using the Ridge model, and of DWIs and T2WIs of MRI using the LASSO model, was validated by 5-fold cross-validation (Fig. 1) of receiver operating characteristic (ROC) analysis. Additionally, we performed the analysis dividing candidate data sets into training cohort and independent validation cohort which is consist of some institutions (Fig. 2). Three institutions out of 13 institutions included more than 20 patients, and one of these institutions was selected as the test cohort and the rest 12 institutions were used in the model development. This procedure was repeated three times by changing the assignment of the test cohort. In the model development, we performed a 5-fold cross validation and prediction accuracy was measured in the test cohort by averaging prediction probability.

The net reclassification improvement (NRI) and integrated discrimination improvement (IDI) statistical test, and concordance index (C-index) were employed to compare the predictive performance between the clinical model and the combined model of clinical information plus radiomics features. We used the Statistical Package for Social Sciences (International Business Machines Corporation, NY) and R ver. 3.6.1 for analyses.

## RESULTS

### Treatment outcomes

The median follow-up period of surviving and lost-to-follow-up patients was 60 months (range, 19–96 months). Twenty-five patients (13.9%) died, all of them died of cervical cancer. The 3-year overall survival rate was 91.1%. The 3-year local control rate of was 94.3%. The 3-year in-field recurrence-free survival rate was 89.8%. Thirty-eight patients developed OFR within three years after the initiation of CCRT. The 3-year OFR-free survival rate was 81.7%. Twenty-one patients had para-aortic lymph node metastases at the initiation of CCRT, and 12 out of 21 (57.1%) developed OFR. Para-aortic lymph node metastasis was a statistically significant prognostic factor of OFR-free survival in univariate analysis, and in multivariate analyses (Table 3). However, there were 159 patients who were para-aortic lymph node metastasis-free at the initiation of CCRT and 26 of these patients (16.4%) developed subsequent OFR. Twenty-six out of 38 (68.4%) patients who developed OFR after CCRT were negative for para-aortic lymph node metastasis at the initiation of treatment.

**Table 3:** Prognostic variables of out-of-field recurrence-free survival

Variables		Number of patients	3-year out-of-field recurrence- free survival (%)	Log-rank test *p*-value	Cox proportional hazards model *p*-value
Age (years)	≦56	98	80.6	0.287	0.673
	>56	82	82.9		
Performance status	0, 1	177	81.9	0.492	0.812
	2	3	66.7		
Histology	Squamous cell carcinoma	167	82.6	0.28	0.948
	Others	13	69.2		
Maximum diameter of primary tumor	≦5.1 cm	90	85.6	0.132	0.557
	>5.1 cm	90	77.8		
Volume of primary tumor	≦33 ml	90	86.7	0.065	0.656
	>33 ml	90	76.7		
Pelvic lymph node metastasis	Yes	68	67.6	<0.001	0.392
	No	112	90.2		
No. of pelvic lymph node metastasis	≧3	19	52.6	0.001	0.977
	0–2	161	85.1		
Common iliac lymph node metastasis	Yes	19	57.9	0.010	0.247
	No	161	84.5		
Para-aortic lymph node metastasis	Yes	21	42.9	<0.001	0.003
	No	159	86.8		
Whole pelvic irradiation, total dose	≦50 Gy	136	86.8	0.006	0.112
	>50 Gy	44	65.9		
Boost therapy to lymph node metastasis	Yes	51	68.6	0.002	0.702
	No	129	86.8		
Brachytherapy, total dose at point A	≦ 24.2 Gy	90	86.7	0.124	0.285
	>24.2 Gy	90	76.7		
Concurrent chemotherapy	Weekly CDDP	133	83.5	0.365	0.857
	Others	47	76.6		

### Out-of-field recurrence prediction

**Fig. 3:**
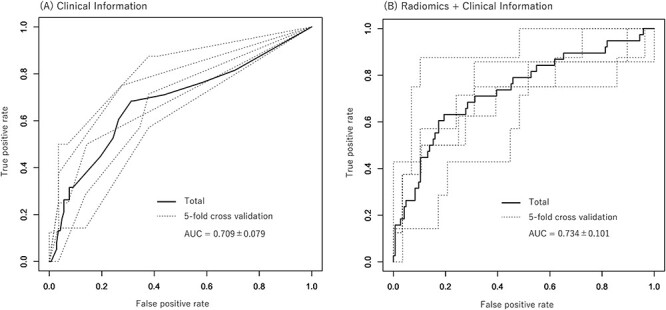
(A) ROC analysis for OFR prediction using the clinical information (status of the para-aortic lymph node metastasis). The dotted line shows the individual results of the 5-fold cross validation, the solid line shows the result of aggregating the five results into one. (B) The ROC analysis for OFR prediction using a combination of the clinical information and engineering image features for DWI of pretreatment MRI with z-score normalization.

**Fig. 4:**
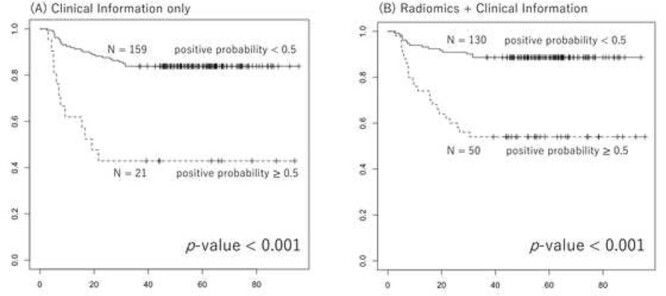
OFR free survival curves of patients divided into two groups with a positive probability of 0.5 calculated by analyses using clinical information (A) and combination of clinical information and ragiomics (B).

**Fig. 5:**
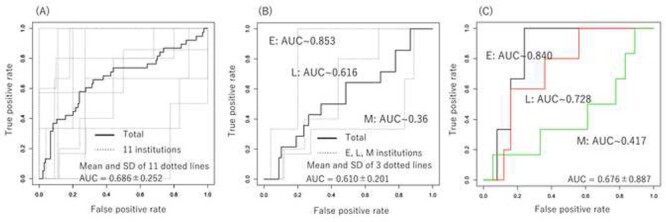
The ROC analysis of LASSO model for DWI using z-score normalization with classification according to the institution. (A) Test results for each of the 11 institutes except for institutes H and J. Let one institute be the test data and the rest be the training data. However, since there were no OFR in H and J, so they are always used as training data. (B) Test results when one of the three institutes with the largest number of patients (E, L, and M) was used as test data. (C) The results of the test data evaluated by the average of five models generated by 5-fold cross validation of the remaining data when one of the three institutes with the largest number of patients (E, L, and M) was used as test data.

We used the pretreatment status of para-aortic lymph node metastasis, the only significant prognostic clinical factor for OFR-free survival, as a clinical factor to predict OFR. Fig. 3(A) shows the results of the ROC analysis for OFR prediction using the Ridge model that used the pretreatment status of para-aortic lymph node metastasis. The dotted line shows the individual results of the 5-fold cross validation, while the solid line shows the aggregate results. The mean (± standard deviation [SD]) area under the curve (AUC) obtained from the ROC analysis of the five dotted lines was 0.709 ± 0.079 (the mean accuracy, sensitivity and specificity was 0.708, 0.736 and 0.607, respectively). The mean (± SD) AUC of the ROC analysis of the LASSO model for DWIs of pretreatment MRI with z-score normalization was 0.677 ± 0.093. Five cross-validations by LASSO for DWI detected 12 features (HHHRLV, HHLSkewness, HLHGCorrelation1, HLHSZLGE, HLHSkewness, HLLLGZE, HLLGCorrelation, LLHLZE, LLHMean, ROIBusyness, Max3Ddiameter, LLLVariance) as significant predicters for OFR four times and 10 features (HHHZLV, HLHLZLGE, HLLGLV, HLLGCorrelation1, LHHMean, LHLRLV, LLLRLV, ROIEnergy, ROILZHGE, SpheDisproportion) five times. The mean (± SD) AUC of the ROC analysis of the LASSO model for T2WIs of pretreatment MRI with z-score normalization was 0.602 ± 0.104. Fig. 3(B) shows the ROC analysis for OFR prediction by a combination of the pretreatment status of para-aortic lymph node metastasis and engineering image features for DWIs of pretreatment MRI with z-score normalization. The mean AUC (± SD) of the ROC analysis of the five dotted lines improved to 0.734 ± 0.101 (the mean accuracy, sensitivity and specificity was 0.769, 0.812 and 0.596, respectively) by combining the two methods. The NRI value (95% confidence interval: CI, *p*-value) and the IDI value in comparison of the ROC analysis results between clinical information plus radiomics features and clinical information only was −0.4375 (−0.7068–0.1682, 0.00145) and − 0.208 (−0.3323–0.0837, 0.00104). The C-index analysis to compare the predictive performance between the clinical model and the combined model of clinical information plus radiomic features showed 0.735 pm 0.048 (*p*-value <0.001) of C-index. Fig. 4 shows OFR free survival curves of patients divided into two groups with a positive probability of 0.5 calculated by analyses using clinical information and combination of clinical information and radiomics. There was a significant difference between the two groups in both analyses. Fig. 5 shows the results of the ROC analysis of LASSO model for DWI using z-score normalization with classification according to the institution. The AUC results were: 0.840, 0.72 and 0.417, for E, L and M institution (the mean accuracy, sensitivity and specificity was 0.821, 0.767 and 0.583, 0.88, 0.84 and 0.722, 0.333, 0.4 and 0.167, respectively). The average is compatible with that from the five-fold cross validation where no institution information was used to divide to the five cohorts. The ROC analysis using the other models than LASSO for OFR prediction by a combination of the pretreatment status of para-aortic lymph node metastasis and engineering image features for DWIs of pretreatment MRI with z-score normalization showed that no model had a higher AUC value than the LASSO model. The mean (± SD) AUC of the ROC by Ridge model was 0.702 ± 0.111 (the mean accuracy, sensitivity and specificity was 0.753, 0.834 and 0.439, respectively). The mean (± SD) AUC of the ROC by Naïve bayes (Gini) model was 0.589 ± 0.154 (the mean accuracy, sensitivity and specificity was 0.758, 0.876 and 0.307, respectively). The mean (± SD) AUC of the ROC by Random Forest (Gini) model was 0.676 ± 0.887 (the mean accuracy, sensitivity and specificity was 0.736, 0.848 and 0.311, respectively) (Supplementary Fig. 1). This may suggest that simple multi-variate model with an embedded method to a feature selection, such as, the LASSO would be appropriate for a limited number of samples. The ROC analysis using the LASSO model for OFR prediction by a combination of the pretreatment status of para-aortic lymph node metastasis and engineering image features for DWIs of pretreatment MRI with two types of image normalization, min–max normalization and z-score normalization showed that AUC value (mean ± SD) was slightly higher with minimum–maximum normalization (0.747 ± 0.093) than z-score normalization (0.734 ± 101), though no statistical significant was found (Supplementary Fig. 2).

## DISCUSSION

While CCRT for stage IIB (FIGO 2008) cervical cancer is expected to provide high local control rates, a persistent challenge for improving outcomes is in determining how to control OFR after CCRT. Control of OFR is key to further improving treatment outcomes. Indications for adjuvant chemotherapy determined based on accurate predictions of the risk of subsequent OFR would increase the chance of preventing subsequent OFR. As this study and previous studies [1, 17] have shown, para-aortic lymph node metastasis is a useful clinical factor for predicting subsequent OFR. However, in this study, 68.4% of patients who developed OFR after CCRT were negative for para-aortic lymph node metastasis at the initiation of treatment, and 16.4% of para-aortic lymph node metastasis-negative patients developed subsequent OFR, and the AUC for OFR prediction using the status of para-aortic lymph node metastasis was 0.709. We thus need an additional biomarker that can be revealed from current routine examinations in order to improve the accuracy of OFR prediction. Creating a highly accurate predictive model that combines clinicopathological information and other biomarkers would lead to a breakthrough in personalized medicine.

Radiomics is a method of extracting features from specific areas of any image, such as those obtained from computed tomography (CT), ultrasound (US), MRI and PET/CT, exhaustively via the use of a computer and has received much attention in recent years. Sanduleanu *et al.* [5] published a systematic review on tumor biology utilizing a radiomics quality score, and reported that all but two studies (n = 39) revealed that radiomics features derived from US, CT, PET and/or MRI were significantly associated with one or several specific tumor biologic substrates, from somatic mutation status to tumor histopathologic grading and metabolism. In the present study, we detected 12 features as significant predictors of OFR four times and 10 features five times in five cross-validations by LASSO. These image features are information derived from the intensity of the voxels and their arrangement, and may reflect the environment of cancer cells at the molecular level. They have a potential to show the features of cancer cells favoring lymphatic or hematologic metastasis, which are not detectable by conventional imaging diagnosis. We think that the radiomics approach can be one of the promising biomarkers based on the results of this study. In order for biomarkers predicted by radiomics approach to become reasonably understandable to oncologists, reliable evidence needs to be accumulated.

In the field of gynecologic oncology, some studies have reported the usefulness of a radiomics approach to MRI and PET/CT [18]. Some of these were studies investigating the prediction of lymph node metastasis. Wu *et al.* [13] retrospectively investigated the radiomics analysis of multiparametric MRI to evaluate tumor grade, lymphovascular space invasion, and lymph node metastasis of 56 patients with cervical squamous cell carcinoma. The AUC for discriminating the presence of lymph node metastasis ranged from 0.747 to 0.850. Wang *et al.* [2] investigated an MRI-based radiomics nomogram for preoperatively predicting pelvic lymph node metastasis in 96 patients with early-stage cervical cancer. The radiomics signature for T2WIs, DWIs and joint T2WIs and DWIs yielded an AUC of 0.844, 0.870 and 0.909, respectively, in the validation cohort. The radiomics nomogram based on joint T2WIs and DWIs demonstrated an improved predictive ability for pelvic lymph node metastasis. Li *et al.* [19] investigated the values of ^18^F-FDG-PET radiomics features combined with vascular endothelial growth factor (VEGF) expression in predicting pelvic lymphatic metastasis in 94 patients with early-stage cervical squamous cell carcinoma. The predictive performance of the ^18^F-FDG-PET radiomics analysis for lymphatic metastases in the validation data set presented by ROC was an AUC of 0.757. The combination of radiomics features and VEGF expression had a significantly superior predictive value (AUC = 0.878).

Compared to the previously published studies, the significance of efficacy of radiomics analysis obtained from the present study stayed at a limited level. One of the possible reasons for this may be that the present study was performed with a multi-institutional setting. The type of disease, stage and treatment method can be the same, but the devices providing the image information cannot be unified. We tried a different method of assigning test and training data than in the single-institute study. The results of the analysis dividing into institution cohorts implies that models trained on data from one institution may have difficulty in analyzing data from other institutions due to the multi-institutional difference such the imaging protocol, machine and so on. For the image feature extraction, differences in MRI hardware and scan protocol and the volume of interest for segmentation may affect the radiomics features [12, 14, 20]. Therefore, it was necessary to normalize the images taken by different MRI models. We performed image normalization using maximum and minimum matching and z-score matching, and those results showed a small deviation. The optimal method to normalize images of various characteristics taken by various modalities was not established. The development of proper image normalization is an important issue because models can only be popularized if they can handle a variety of images obtained using different equipment. Establishing a proper image normalization and a method for image data acquisition suitable for computer analyses in a multicenter study is expected to improve the accuracy of predictions. Model optimization is an ongoing issue. Importantly, we highlight the need to develop models with higher prediction accuracy as we continue to increase the number of patients.

The limitation of the present study lies in its retrospective design and the limited sample size. A prospective study that proceed in parallel with continuous refinement of the predictive model is necessary.

In conclusion, para-aortic lymph node metastasis was a statistically significant prognostic factor of OFR after CCRT for cervical cancer in stage IIB (FIGO 2008). Combining MRI radiomics with pretreatment status of para-aortic lymph node metastasis improved the accuracy of predicting OFR.

## Supplementary Material

Supplementary_Figure_1_rrab104

Supplementary_Figure_2_rrab104

## PRESENTATION AT A CONFERENCE

The present study was presented at the 33^rd^ annual meeting of the Japanese Society for Radiation Oncology.
