# Supplementary figures and images for: Prediction of out-of-field recurrence after chemoradiotherapy for cervical cancer using a combination model of clinical parameters and magnetic resonance imaging radiomics: a multi-institutional study of the Japanese Radiation Oncology Study Group
Source: J Radiat Res. 2021 Dec 3;63(1):98–106. doi: 10.1093/jrr/rrab104 (PMC8776693; doi:10.1093/jrr/rrab104)

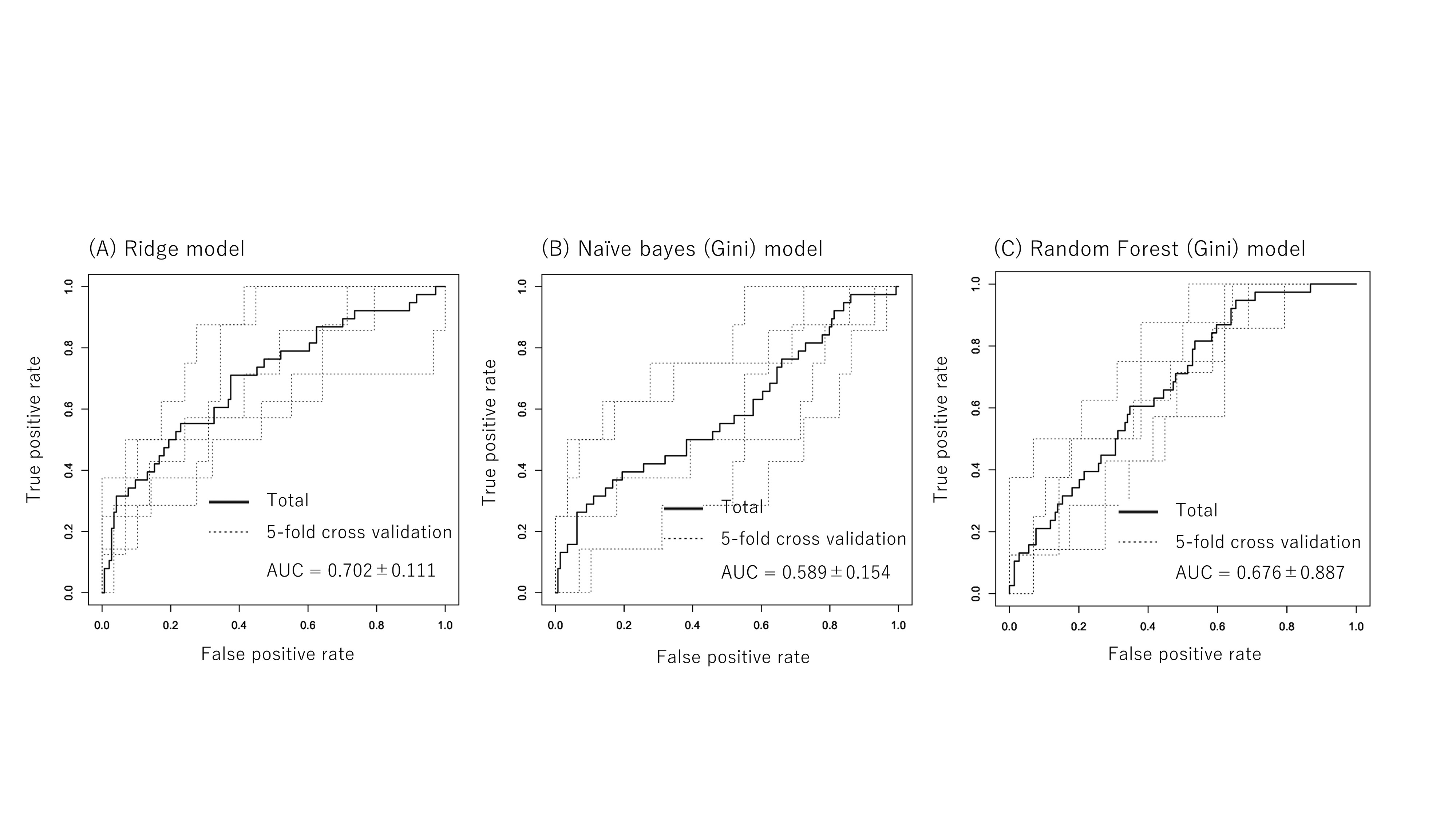

Supplement: Supplementary_Figure_1_rrab104 [file supplementary_figure_1_rrab104.jpeg]

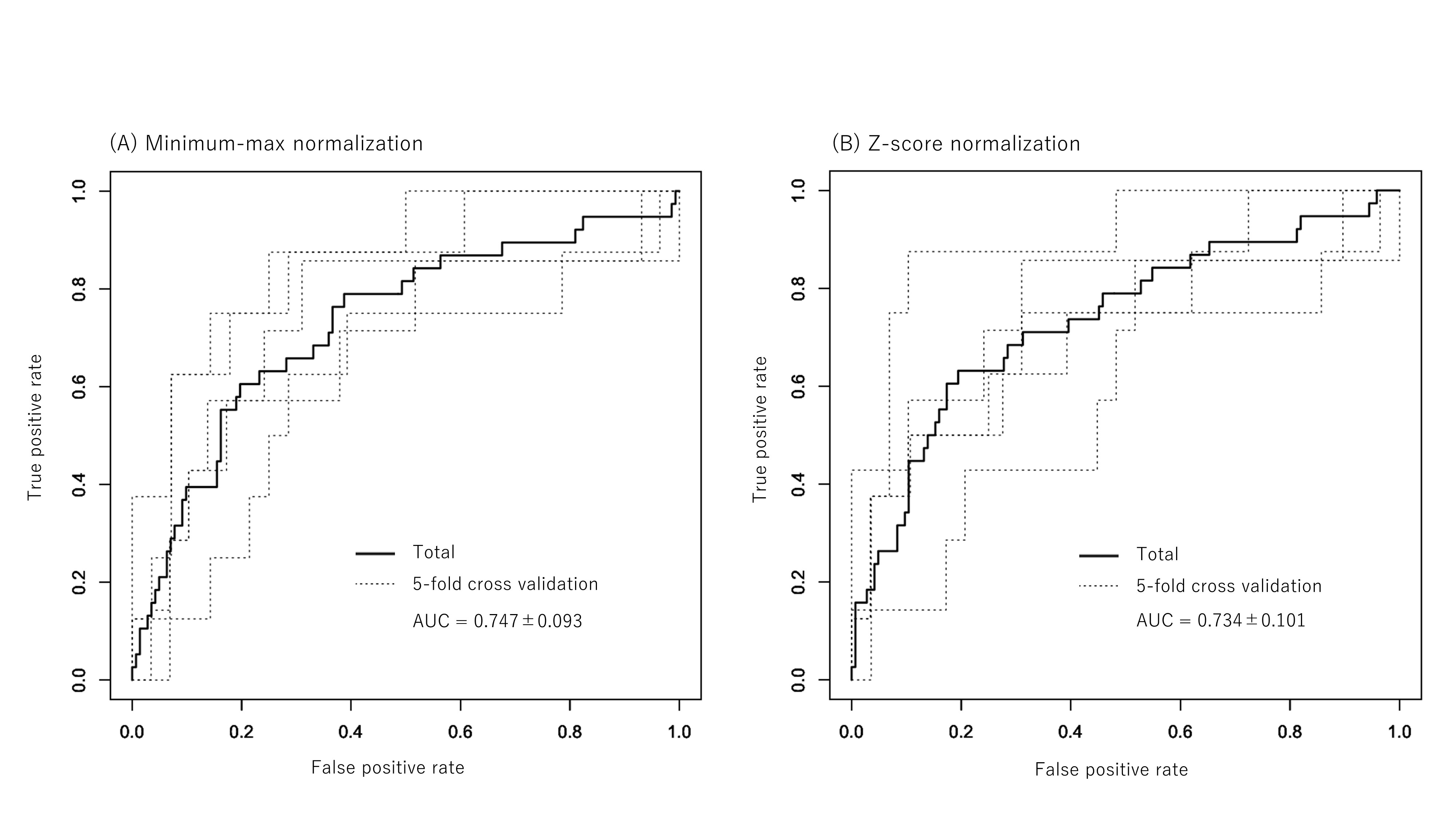

Supplement: Supplementary_Figure_2_rrab104 [file supplementary_figure_2_rrab104.jpeg]
